# Typhoid Fever

**Published:** 1912-10

**Authors:** 


Typhoid Fever.—In a large majority of cases of typhoid fever there is undoubtedly an intestinal lesion, but other organs are also affected. In a few cases post-mortem examination reveals no lesion whatsoever in the alimentary tract. Typhoid fever differs from some of the infectious diseases in that during its course the entire body is exposed to a specific bacillus and that the lesions are, therefore, really several fold. Many physicians do not admit this fact and speak of and treat enteric fever as if it were an infection confined to the intestinal canal. In typhoid fever, on the other hand, the patient may be seriously sick with a non-enteric typhoid and yet have an intestine totally free from the typhoid bacilli and from any of the intestinal lesions of the disease. The report from pathologists show that many cases are now on record in which typhoid fever was present and in which no intestinal lesion was found. If the disease is an infection involving various organs of the economy, the treatment which only has in view the lesions found in the intestinal canal will be inadequate to meet successfully the patient's condition; consequently a close and careful study should be made of any suggestive cause.

In the treatment of typhoid fever the patient should be in an aseptic, well-ventilated, light and cheerful room. He should have water at stated intervals. It is a great mistake to neglect this, as when a patient is unconscious he should have water and, of course, does not then ask for it.

The medical treatment of enteric fever is largely symptomatic, the patient suffering from the infection produced by the typhoid bacillus. The body is necessarily affected by splenic toxemia; the intestinal glands and other organs are involved. Prominent among the latter symptoms are emaciation and malnutrition, and this should be combated by a food which will not overtax the digestive system, and will at the same time supply every element of nutrition. Bovinine is ideally indicated as a food. From the onset, antiseptics are indicated and should be administered more or less throughout the entire course of the disease; but, most of all, keep the patient's temperature down by sponge baths, and the strength and nutrition as near normal as possible.

---

The Amendment to the Pure Food and Drug Law, which it was thought would put an end to the unfounded claims of the patent medicine makers, will be ineffective, according to Dr. Wiley. The amendment says that the claims as to curative effects, printed on the labels of the bottles, shall not be false and fraudulent, and this, says Dr. Wiley, will be interpreted as meaning that they may be either false or fraudulent, but must not be both. It is possible to imagine a lawyer contending that the fake statements made by his client were believed by the latter to be true and were therefore not fraudulent. Dr. Wiley says: "The committee had before it a perfectly just amendment drawn in no uncertain terms that not only covered false and fraudulent claims on the labels but also the same claims printed on billboards or in advertising matter, thus striking a mortal blow at the fakes and frauds which have been a curse to the public. In securing the passage of the Sherley amendment the venders of fraudulent preparations have won a complete victory. It appears that Congress has only given them new life, enabling the roots of fraud and corruption to sink deeper into the soil of legislation, protected of the vested interests but not of the public welfare."—*New York Medical Journal.*

